# Cross-cultural analysis of the stigmatising attitudes of psychiatrists across Europe and measurement invariance of the Opening Minds Stigma Scale for healthcare providers

**DOI:** 10.1192/j.eurpsy.2022.539

**Published:** 2022-09-01

**Authors:** D. Ori, P. Szocsics, T. Molnar, S. Rozsa, M. Wallies, O. Kazakova, L. Bankovska-Motlova, S. Boivin, S. Raaj, I.M. Overgaard Ingeholm Klinkby, C. Cabacos, A.T. Pereira, S. Matheiken, S. Kakar, S. Greguras, J. Maslak, N. Nechepurenko, K. Kotsis, H. Yilmaz Kafali, A. Mirkovic, P. Rus Prelog, K. Bruna, K. Guevara, R. Strumila, S. Mörkl, M. Abdulhakim, E.A. Carbone, A. Panayi, I. Ivanović, E. Dashi, G. Grech, M. Vircik, F. Schuster, J. Soler-Vidal, E. Pomarol-Clotet, G. Ahmadova, A. Hargi, H. Kisand, N. Grinko, Z. Gyorffy

**Affiliations:** 1 Heim Pál National Pediatric Institute, Department Of Mental Health, Budapest, Hungary; 2 Semmelweis University, Department Of Psychiatry And Psychotherapy, Budapest, Hungary; 3 Petz Aladar County Hospital, Department Of Psychiatry, Budapest, Hungary; 4 Károli Gáspár University of the Reformed Church, Department Of Health Psychology, Budapest, Hungary; 5 Psychiatrische Klinik Clienia Littenheid, Psychiatrische Klinik Clienia Littenheid, Sirnach, Switzerland; 6 Psychiatric Clinic of Minsk City, Department Of Psychiatry, Minsk, Belarus; 7 Charles University, 3rd Faculty Of Medicine, Prague, Czech Republic; 8 EPSM Étienne Gourmelen, Epsm Étienne Gourmelen, Quimper, France; 9 Mater University Hospita, Department Of Liasion Psychiatry, Dublin, Ireland; 10 Psychiatry- Aalborg University Hospital, Research Unit For Child And Adolescent Psychiatry, Aalborg, Denmark; 11 Faculty of Medicine of University of Coimbra, Institute Of Psychological Medicine, Coimbra, Portugal; 12 Pennine Care NHS Foundation Trust, Pennine Care Nhs Foundation Trust, Oldham, United Kingdom; 13 Erasmus university, Erasmus University, Rotterdam, Netherlands; 14 University Hospital Centre Zagreb, Department Of Psychiatry, Zagreb, Croatia; 15 Institute for Mental Health, Institute For Mental Health, Belgrade, Serbia; 16 The Serbsky State Scientific Center for Social and Forensic Psychiatry, The Serbsky State Scientific Center For Social And Forensic Psychiatry, Moscow, Russian Federation; 17 University of Ioannina, Department Of Psychiatry, Ioannina, Greece; 18 Ankara City Hospital Bilkent, Ankara City Hospital Bilkent, Ankara, Turkey; 19 Children’s hospital Ljubljana, Children’s Hospital Ljubljana, Ljubljana, Slovenia; 20 University Psychiatric Clinic Ljubljana, University Psychiatric Clinic Ljubljana, Ljubljana, Slovenia; 21 Psychiatric Hospital Gintermuiza, Psychiatric Hospital Gintermuiza, Jelgava, Latvia; 22 Military Medical Academy, Department Of Psychiatry, Sofia, Bulgaria; 23 Vilnius University, Psychiatric Clinic, Vilnius, Lithuania; 24 Medical University of Graz, Department Of Psychiatry And Psychotherapeutic Medicine, Graz, Austria; 25 Vrije Universiteit Brussel, Department Of Psychiatry, Brussels, Belgium; 26 University Magna Graecia of Catanzaro, Department Of Psychiatry, Catanzaro, Italy; 27 Freelancer, Freelancer, Nicosia, Cyprus; 28 Clinical Centre of Montenegro, Clinic For Psychiatry, Podgorica, Montenegro; 29 Xhavit Gjata Hospital, Xhavit Gjata Hospital, Tirane, Albania; 30 Mount Carmel Hospital, Mount Carmel Hospital, Attard, Malta; 31 Psychiatric Hospital Michalovce, Psychiatric Hospital Michalovce, Michalovce, Slovak Republic; 32 Technischen Universität München, Klinikum Rechts Der Isar Der Technischen Universität München, Munich, Germany; 33 Fidmag Research Foundation, Fidmag Research Foundation, Barcelona, Spain; 34 City Hospital N15, , Department Of Psychiatry, Baku, Azerbaijan; 35 University of Tartu, University Of Tartu, Tartu, Estonia; 36 Chernivtsi Reginal Mental Hospital, Chernivtsi Reginal Mental Hospital, Chernivtsi, Ukraine; 37 Semmelweis University, Institute Of Behavioural Sciences, Budapest, Hungary

**Keywords:** mental health related stigma, measurement invariance, attitudes of psychiatrists, cross-cultural analysis

## Abstract

**Introduction:**

Since the literature investigating the stigmatising attitudes of psychiatrists is scarce, this is the first study which examines the phenomena across Europe. The Opening Minds Stigma Scale for Health Care Providers (OMS-HC) is a widely used questionnaire to measure stigma in healthcare providers towards people with mental illness, although it has not been validated in many European countries.

**Objectives:**

A cross-sectional, observational, multi-centre study was conducted in 32 European countries to investigate the attitudes towards patients among specialists and trainees in general adult and child psychiatry. In order to be able to compare stigma scores across cultures, we aimed to calculate measurement invariance.

**Methods:**

An internet-based, anonymous survey was distributed in the participating countries, which was completed by n=4245 psychiatrists. The factor structure of the scale was investigated by using separate confirmatory factor analyses for each country. The cross-cultural validation was based on multigroup confirmatory factor analyses.

**Results:**

When country data were analysed separately, the three dimensions of the OMS-HC were confirmed, and the bifactor model showed the best model fit. However, in some countries, a few items were found to be weak. The attitudes towards patients seemed favourable since stigma scores were less than half of the reachable maximum. Results allowed comparison to be made between stigma scores in different countries and subgroups.

**Conclusions:**

This international cooperation has led to the cross-cultural validation of the OMS-HC on a large sample of practicing psychiatrists. The results will be useful in the evaluation of future anti-stigma interventions and will contribute to the knowledge of stigma.

**Disclosure:**

No significant relationships.

